# Moxibustion using different habitat moxa floss for moderate to severe primary knee osteoarthritis: study protocol for a three-armed randomized, double-blinded, sham-controlled trial

**DOI:** 10.1186/s13063-018-2794-1

**Published:** 2018-07-27

**Authors:** Huanfang Xu, Hong Zhao, Liping Kang, Shixi Huang, Yin Shi, Wei Su, Mingjuan Han, Wenyan Wang, Chunyan Wang, Yuan Zhang, Lanping Guo

**Affiliations:** 1 Institute of Acupuncture and Moxibustion, China Academy of Chinese Medical Sciences, Beijing, China; 2 Guang’anmen Hospital China Academy of Chinese Medical Sciences, Beijing, China; 3 Shanghai Research Institute of Acupuncture and Meridian, Shanghai, China; 4 Beijing First Hospital of Integrated Chinese and Western Medicine, Beijing, China; 5 State Key Laboratory Breeding Base of Dao-di Herbs, National Resource Center for Chinese Materia Medica, China Academy of Chinese Medical Sciences, Beijing, China

**Keywords:** Moxibustion, Moxa floss, Habitat, Knee osteoarthritis, Randomized controlled trial, Study protocol

## Abstract

**Background:**

According to the traditional Chinese medicine theory, moxa floss is the best material for moxibustion; the effect of moxibustion is closely related to the habitats of moxa floss, among which Qichun County, Hubei Province, China, is considered as the genuine origin. However, this view has not been validated by clinical studies. Moxibustion has been proven effective in alleviating pain and improving physical function and quality of life for patients with knee osteoarthritis (KOA). This trial aims to determine whether the habitat of moxa floss contributes to the effect of moxibustion and to validate the effectiveness of moxibustion for KOA.

**Methods:**

This is a three-armed, randomized, double-blinded, sham-controlled trial. A total of 350 patients with moderate to severe primary KOA will be randomly allocated to groups A, B, or C with a 2:2:1 ratio. Moxa stick moxibustion using moxa floss from different habitats will be applied in two experimental groups: group A, moxa floss from the habitat of Qichun County, Hubei Province, China; and group B, moxa floss from the habitat of Nanyang County, Henan Province. Group C will use non-moxa floss for sham moxibustion as control. Patients will be treated for 20 min per session, for three sessions per week for 2 weeks, and followed up for 4 weeks. The primary outcome will be the change from baseline in the pain score of the Western Ontario and McMaster Osteoarthritis Index (WOMAC) at week 2. Secondary outcomes will include a change in the WOMAC pain score at week 6; the visual analogue scale for knee pain, the total WOMAC score, the WOMAC stiffness score, the WOMAC function score, the patient global assessment, and the responder criteria at weeks 2 and 6. Adverse events will be assessed throughout the study.

**Discussion:**

This trial will help to identify the effectiveness of moxibustion for KOA and whether the habitat of moxa floss contributes to the effect of moxibustion.

**Trial registration:**

Acupuncture-Moxibustion Clinical Trial Registry: AMCTR-IOR-16000007. Registered on 29 February 2016.

**Electronic supplementary material:**

The online version of this article (10.1186/s13063-018-2794-1) contains supplementary material, which is available to authorized users.

## Background

Knee osteoarthritis (KOA) affects a large proportion of the population worldwide, especially the elderly, and leads to pain and disability. The prevalence of symptomatic KOA reached 10.3% and 5.7% in women and men aged 45 years and over, respectively, in China [1]. In the age group of 70 years and older, Chinese women showed a higher prevalence of KOA than those in the Framingham osteoarthritis (OA) study [2, 3]. KOA, together with hip OA, being the 11th highest contributor to global disability and 38th highest in disability-adjusted life years, has constituted a major health burden globally [4]. However, the treatment of KOA is still far from satisfactory. Most guidelines have recommended a multimodal pharmacologic and non-pharmacologic approach until total knee replacement is indicated [5–7]. The predominant pharmacologic therapy, mainly including acetaminophen, nonsteroidal anti-inflammatory drugs (NSAIDs), and intra-articular corticosteroid injections, can effectively relieve KOA symptoms. However, these therapies always carry undesired gastrointestinal, cardiovascular, renal, and hepatic side effects, especially with long-term use [8–10]. Hence, many patients begin to seek help from complementary and alternative medicine, among which moxibustion is one of the most extensively used therapies [11]. Although strong evidence is needed, meta-analyses indicate that moxibustion is not statistically different from oral drugs, and is superior to usual care and sham moxibustion in alleviating pain and improving physical function and quality of life [12]. Moreover, most adverse events (AEs) caused by moxibustion can heal without medical care [12].

Moxibustion is a traditional Chinese medicine (TCM) therapy involving ignited material to apply heat to certain acupuncture points or areas of the body surface for curing disease through the regulation of the function of meridians and visceral organs [13]. Among the various materials used for moxibustion, moxa floss, a cotton-like material made from dried leaves of Artemisia argyi (a plant commonly known as moxa or mugwort) [13], has survived the test of long-term medical practice and become the most recognized version. Since mugwort is widespread throughout China, there are many habitats for commercially available moxa floss, among which Qichun County, Hubei Province, and Nanyang County, Henan Province become the two largest producers. According to the classical TCM theory, the habitat of moxa floss is crucial to the effect of moxibustion, and the best habitat is granted to Qichun County, Hubei Province. It has been reported that dried leaves of Artemisia argyi native to Qichun County, Hubei Province, produce a higher content of volatile components and a larger output of combustion heat release than those native to other habitats in China [14, 15]. However, this does not imply a similar clinical effect of moxibustion. Though moxa floss native to Qichun County, Hubei Province has been long believed to be the optimal moxibustion material by TCM practitioners, so far there are no clinical studies comparing the moxibustion effect among moxa floss from different habitats. The primary objective of this randomized controlled trial is to compare the effects of moxibustion using moxa floss from Qichun County, Hubei Province (Qichun moxa floss) versus those from Nanyang County, Henan Province (Nanyang moxa floss) in alleviating knee pain for patients with KOA, thus to determine whether the habitat of moxa floss contributes to the effect of moxibustion. The results of this study will also help to validate the effectiveness of moxibustion for KOA via the comparison between moxibustion and sham moxibustion.

## Methods

### Study design

This is a multicenter, three-armed, randomized controlled trial. The trial will be conducted at four hospitals in China: the Institute of Acupuncture and Moxibustion China Academy of Chinese Medical Sciences (CACMS), the Guang’anmen Hospital CACMS, the Shanghai Research Institute of Acupuncture and Meridian, and the Beijing First Hospital of Integrated Chinese and Western Medicine, between 1 March 2016 and 30 December 2017. This protocol is in accordance with the principles of the Declaration of Helsinki, and has been approved by the ethics committee of each participating hospital. Prior to enrollment, patients will give their written informed consent after acquiring detailed information of the study from the staff responsible for recruitment.

**Fig. 1:**
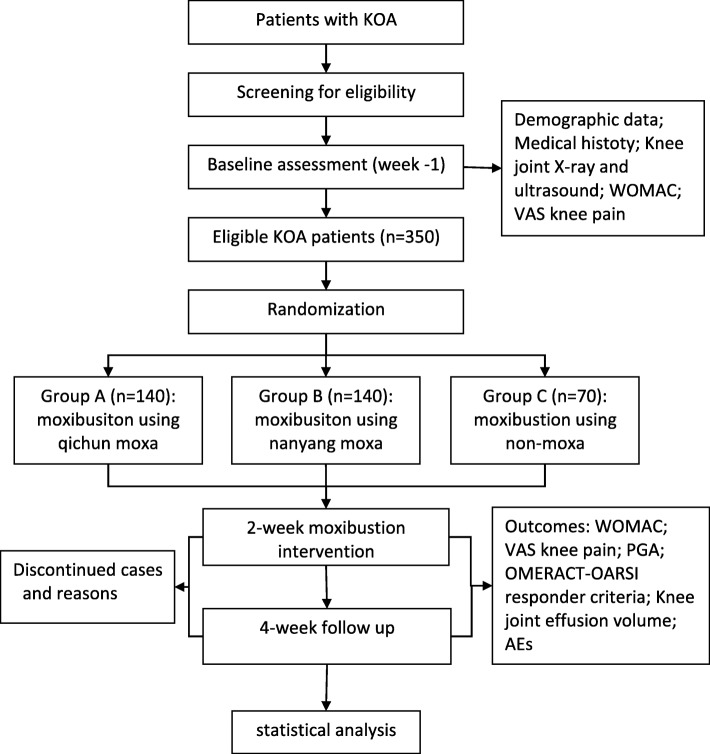
Trial flow chart

**Fig. 2:**
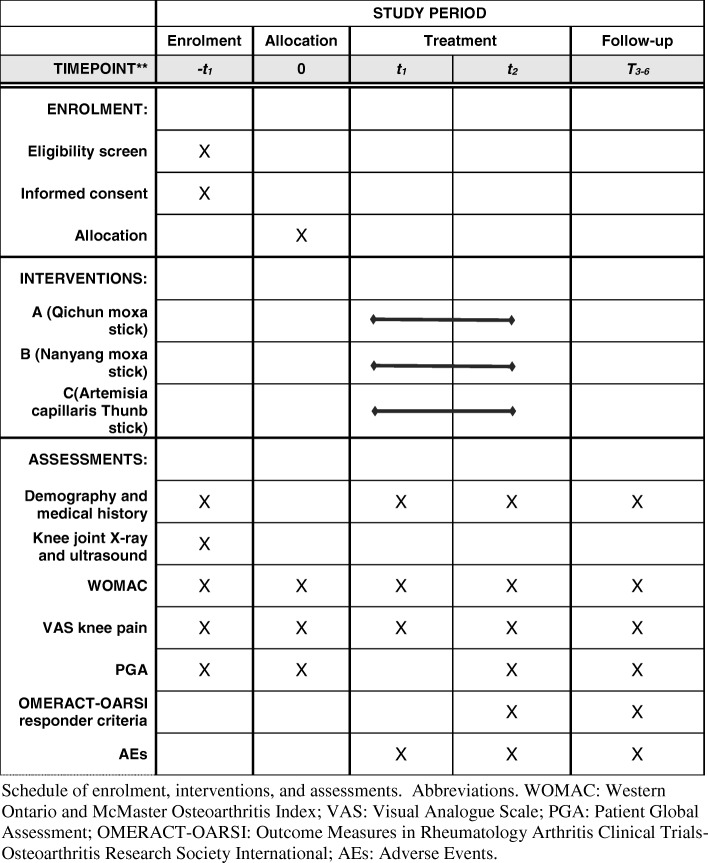
Moxa and non-moxa sticks

Eligible patients will be randomly assigned to receive one of the three kinds of moxibustion treatments: moxibustion using Qichun moxa floss (group A), Nanyang moxa floss (group B), or non-moxa floss (group C) for 2 weeks, and be followed up for 4 weeks (Fig. 1 and Fig. 2). Outcomes will be assessed at baseline, after a 2-week treatment and after a 4-week follow-up. This protocol has been registered at the Acupuncture-Moxibustion Clinical Trial Registry (AMCTR-IOR-16000007), a secondary register platform affiliated to the Acupuncture Clinical Trial Registration Center site and WHO International Clinical Trials Register Platform.

### Participants

This trial plans to recruit 350 participants with moderate to severe KOA from the outpatients of the acupuncture and moxibustion clinics of the four hospitals via poster, website, and WeChat.

Participants will be included if they fulfill the following criteria: (1) aged 50–80 years; (2) diagnosed as primary KOA according to the American College of Rheumatology clinical and radiographic criteria [16] (knee pain; osteophytes; and at least one of the three following conditions: (a) age > 50 years; (b) stiffness < 30 min; (c) crepitus); (3) radiological assessment of KOA rated as grade 2 or 3 on the Kellgren-Lawrence Grading Scale [17] (grade 2: definite osteophytes and possible joint space narrowing on anteroposterior weight-bearing radiograph; grade 3: multiple osteophytes, definite joint space narrowing, sclerosis, and possible bony deformity); and (4) an overall score of > 49 to ≤ 72 on the Western Ontario and McMaster Universities Osteoarthritis Index (WOMAC) (0–96) [18]; that is moderate to severe KOA.

Participants will be excluded with any of the following items: (1) secondary KOA; (2) significant trauma or surgery history in the target knee joint within 1 year, or severe knee joint deformity (varus or valgum with an angle of at least 8 degrees); (3) recent intra-articular injection with glucocorticoids < 3 months previously or hyaluronic acid < 2 weeks previously, or acupuncture or moxibustion treatment within 2 weeks; (4) symptomatic hip OA or patellofemoral arthritis homolateral to the target knee; (5) inflammatory diseases including rheumatoid arthritis; (6) acute meniscus injury or ligamentous injury or rupture of knee joint; (7) knee joint peripheral tumor, tuberculosis or spontaneous osteonecrosis of the knee; (8) taking paracetamol, an anti-inflammatory agent or an analgesic for other diseases; (9) uncontrolled hypertension or diabetes, tumor or serious cardiovascular, cerebral, lung, liver, spleen, kidney, hematopoietic, hemorrhagic or psychiatric disease; (10) scar diathesis or sensory disorder; (11) pregnant or lactating women; (12) refusal to sign informed consent.

### Randomization and masking

A block randomization, stratified by center, will be applied in this trial. Eligible participants will be randomly assigned to group A, B, or C with a 2:2:1 ratio. The central randomization system of the Institute of Basic Research in Clinical Medicine, CACMS will be used in this trial. The independent research assistants in each center will get a random number and group assignment by telephone or message from the system.

**Fig. 3:**
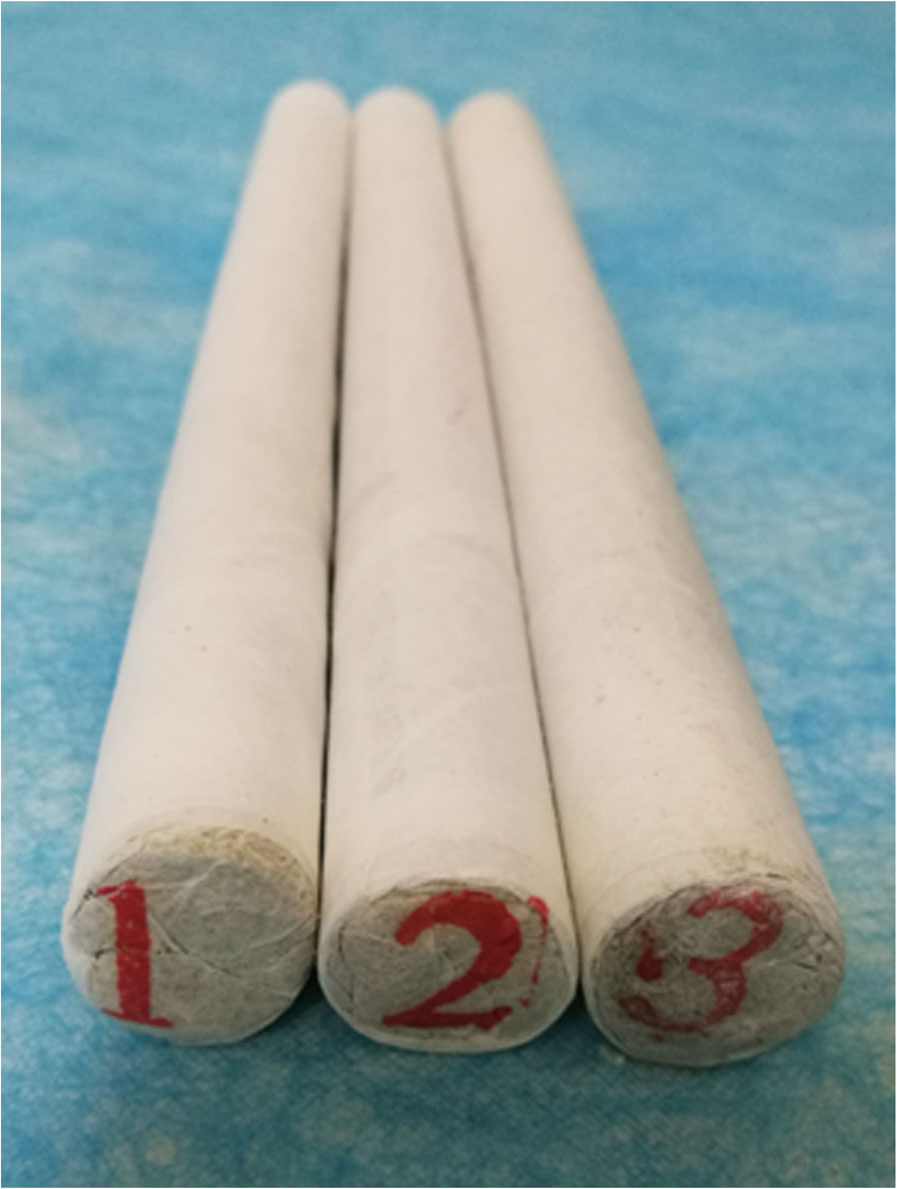
Moxibustion apparatus. **a** base, **b** adjustable holder, **c** flexible tube, **d** clamp

In this trial, moxa stick moxibustion, a type of moxibustion, will be applied. Moxa floss with Artemisia argyi leaves stored for 5 years from two habitats, Qichun County, Hubei Province, China (a habitat recognized by TCM theory and the top producer for commercially available moxa products) and Nanyang County, Henan Province, China (another top producer for commercially available moxa products), will be processed into moxa sticks named as A (Qichun moxa stick) and B (Nanyang moxa stick). For the sham control, a kind of non-moxa stick, named as C, will be made from the floss of Artemisia capillaris Thunb (a plant belonging to the same family and genus with Artemisia argyi), which presents a similar color and inflammability with moxa floss. The moxa and non-moxa sticks used in this trial will be made by the same process with the same specifications (a diameter of 1.75 cm and a length of 20 cm) and appearance, but with different labels by the same company (Nanyang Chinese Medicine Moxa LLB) (Fig. 3). The blindness effect of moxibustion using these three kinds of moxa sticks (Qichun moxa stick, Nanyang moxa stick, and non-moxa stick) has been assessed in both patients and practitioners pre-study. The results showed that patients cannot differentiate moxibustion between any of the three moxa sticks, while practitioners can differentiate non-moxa stick moxibustion from moxa stick moxibustion via brining smell, burning speed and tightness of ashes. However, they cannot distinguish moxibustion using the Qichun moxa stick from those using the Nanyang moxa stick (unpublished data). The practitioners, participants, outcome assessors, and statisticians will be kept blinded to treatment allocation throughout the trial. In this trial, emergency unblinding is not applicable.

### Intervention

**Fig. 4:**
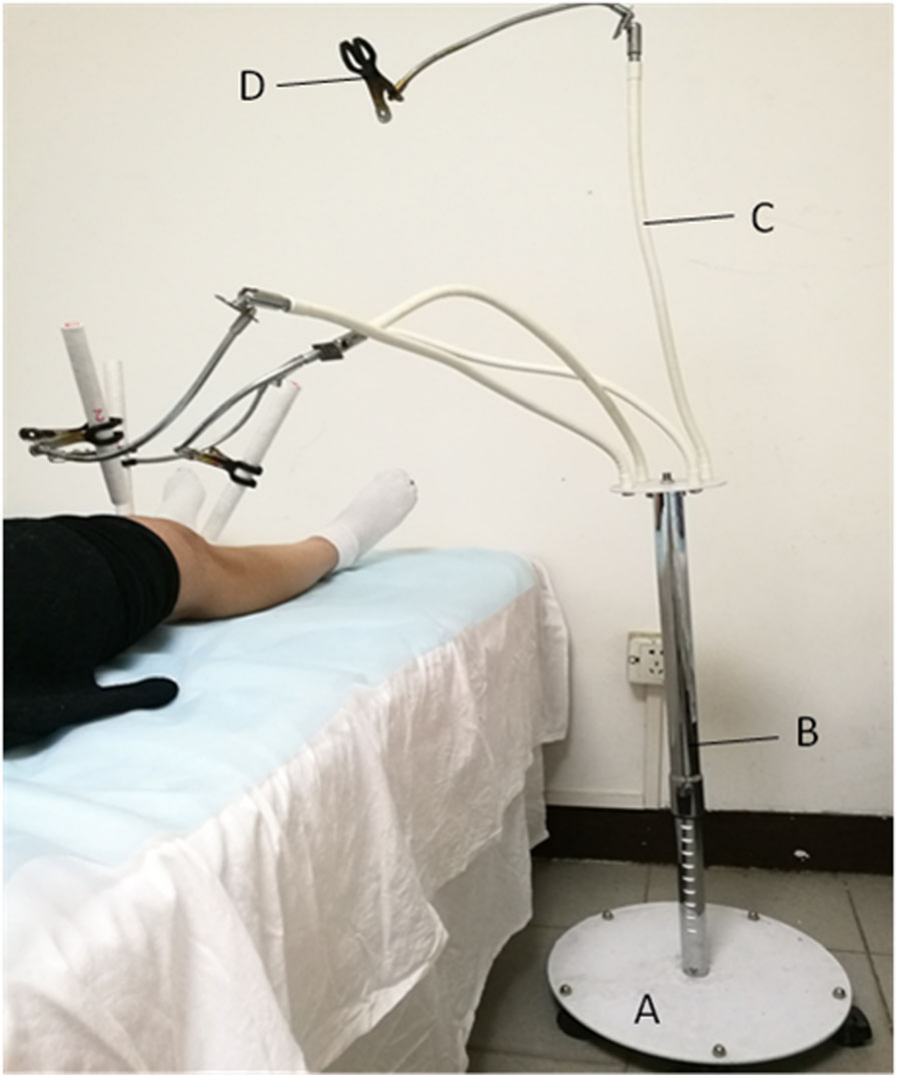
SPIRIT Figure

Patients in each group will receive moxibustion using the corresponding moxa sticks (group A: moxa stick A; group B: moxa stick B; and group C: moxa stick C) on three standard acupoints (ST35, Ex-LE04, and Ex-LE02) of the affected knee 20 min per session, three sessions per week (ideally every other day) for 2 weeks. For patients with both knees affected, treatments will be provided on the knee with more severe baseline pain. The treatment will be conducted at a room temperature of 22–26 °C. A four-head, floor-type moxibustion apparatus, consisting of a base, an adjustable holder and four flexible tubes with clamps, will be used for the implementation of moxibustion (Fig. 4). The three groups will share the same moxibustion procedure as follows: (1) patients will be kept in a supine position with their knees slightly flexed (a soft, rolled blanket may be placed under the knee joints) to avoid hyperextension; (2) the holder of the moxibustion apparatus will be adjusted to a proper height; each of the three used clamps will be fixed to one end of the moxa stick with at least 6 cm left in the other end (named the burning tip); and the flexible tubes will be adjusted to make each burning tip of the moxa stick 2.5–3 cm above each acupoint; (3) the burning tips of the three moxa sticks will be ignited; (4) patients will be required to feel an intolerable causalgia (approximately 40–43 °C) within 5 min. Once this is attained, the height of the burning tip will be quickly adjusted to allow patients to keep a comfortable moxibustion sensation during the rest of the treatment.

Throughout the trial, the patients will be discouraged from receiving drug therapy for KOA, including acetaminophen, NSAIDs, other analgesics, etc. For any unallowed treatment that has already been used, relevant information should be recorded in the patient’s electronic Case Report Form (eCRF) in detail. Medicine used for other diseases will not be forbidden.

### Outcome measures

The primary outcome will be the change in the score on the pain subscale (pain score) of the WOMAC from baseline to week 2.

The WOMAC is a widely used disease-specific measurement for function of hip and/or knee OA [19]. It consists of 24 items divided into 3 subscales addressing severity of joint pain (5 items), stiffness (2 items) and limitation of physical function (17 items), respectively, in the previous 48 h. In this trial, a validated Chinese WOMAC [20] will be used. Each item of WOMAC will be assessed by a 100 mm visual analogue scale (VAS), with a higher score indicating worse symptom severity. Items will be summed for each subscale, resulting in possible ranges as follows: pain, 0–500 mm; stiffness, 0–200 mm and physical function, 0–1700 mm. A total WOMAC score is a sum of the scores for all three subscales, with 2400 mm being the worst possible total score.

Secondary outcomes include the change in the WOMAC pain score from baseline to week 6; the change from baseline to weeks 2 and 6 in knee pain (measured by a 100 mm VAS with higher score indicating more severe pain), the total WOMAC score, the WOMAC stiffness score, the WOMAC function score, and the patient global assessment (PGA) (measured by a 100 mm VAS with a higher score indicating a patient more greatly affected by KOA); and the responder criteria developed by the Outcome Measures in Rheumatology Arthritis Clinical Trials-Osteoarthritis Research Society International (OMERACT-OARSI) criteria [21]. The OMERACT-OARSI responders are defined as patients with (1) at least 50% improvement and an absolute change of at least 20 points in the WOMAC pain score or WOMAC function score; or (2) at least 20% improvement and an absolute change of at least 10 points in any two of the WOMAC pain score, the WOMAC function score, and the PGA.

The change from baseline in skin temperature for each acupoint (ST35, Ex-LE04, and Ex-LE02) at 5 min, 10 min, and 15 min during moxibustion will be assessed using an UT-325 Contact Type Thermometer (Uni-Trend Technology (China) Limited, Guangdong, China). A total of 30 patients will be randomly selected from patients in the Institute of Acupuncture and Moxibustion CACMS, 10 patients per group. The skin temperature for each acupoint will be measured at 5 min, 10 min, and 15 min during moxibustion, three measures for each acupoint per moxibustion session, totaling six sessions of moxibustion. The skin temperature for each acupoint at each corresponding time point is defined as the mean of total skin temperatures measured at the corresponding time point during each of the six moxibustion sessions. For example, the skin temperature for ST35 at 5 min during moxibustion is the mean of total skin temperatures measured at 5 min during each of the six moxibustion sessions.

Adverse events (AEs) will be appropriately monitored, managed, and documented throughout the trial. According to their potential association with moxibustion, the AEs will be categorized by acupuncturists as treatment-related or non-treatment-related within 24 h of their occurrence. AEs related to moxibustion mainly include symptoms of excessive heat: dryness of eye, mouth, throat, or nose, sore throat, eye itching, runny nose, tearing, swelling, and aching of gum, etc.; skin symptoms: empyrosis, rubefaction, blistering, itching sensations, pigmentation, etc.; or other symptoms: general fatigue, dizziness insomnia, drowsiness, constipation, diarrhea, flatus, etc.

### Statistical methods

#### Sample size

There are no previous clinical studies comparing different habitats of moxa floss, or are there studies comparing moxa floss with non-moxa floss for the effect of moxibustion. Based on the results of a study investigating moxibustion versus a heat-insulated placebo moxibustion for KOA [22], we assumed a standard deviation of 10 for group C (sham moxibustion), and 12 for groups A and B (moxibustion). The sample size calculation was based on the formula of one-way analysis of variance (ANOVA) with pairwise comparisons, with 80% power, a two-sided significance level of 5% and a 15% dropout rate. We designed this study to detect: (1) a difference of 5 points in the WOMAC pain score for comparison between moxibustion using different habitat moxa floss; that is groups A and B with an allocation ratio of 1:1; and (2) a minimal clinically important difference of 10 points [23, 24] for comparison between moxibustion (group A or B) and sham moxibustion (group C) with an allocation ratio of 2:1. For the first comparison, 140 participants will be needed for groups A and B; for the second comparison, 45 participants will be needed for group C, and 90 for groups A or B. The larger result from the first comparison was then used to determine the final sample size, that is, 140 participants in groups A and B, respectively, and 70 in group C. The total sample size is 350 participants.

#### Statistical analysis

Data analyses will be performed by a statistician blinded to group assignments using SAS version 9.4 (SAS Institute, Cary, NC, USA). Analysis will be performed by intention-to-treat principle, with all randomly assigned participants included. Missing data will be imputed using the multiple imputation method. The primary outcome will be analyzed using analysis of covariance (ANCOVA) or Kruskal–Wallis H test, as appropriate. The ANCOVA model will include changes in the WOMAC pain score from baseline to week 2 as the dependent variable, treatment as the independent variable of interest, and the baseline WOMAC pain score and study site as covariates; when it is significant (*P* < 0.05), the Tukey–Kramer adjustment will be applied for pairwise comparisons across groups while maintaining 5% type I error rate. In case the Kruskal–Wallis H test is significant (*P* < 0.05), Mann–Whitney *U* test will be applied for pairwise comparison, and the significance level for the type I error will be adjusted by Bonferroni correction at *P* < 0.017.

For secondary outcomes, continuous variables will be assessed using ANOVA or the Kruskal-Wallis H test, as appropriate, with the same multiple comparison methods as the primary outcome; binary categorical variables will be compared using the Cochran–Mantel–Haenszel method adjusted for randomization strata.

### Quality control

The trial protocol is developed after consulting experts of acupuncture and moxibustion, orthopedics, statistics, and methodology. All staff will be required to receive training on the trial protocol, participant selection, moxibustion intervention, effect assessment, the usage of the central randomization and data management systems, and the methods to fill out the materials of the trial (e.g. the case report forms), before participating in the trial. Data management will be performed by the Institute of Basic Research in Clinical Medicine, CACMS. Double data entry and strict data audit will be applied. Personal information about patients will be coded using patient identification numbers. Data in the eCRF will be completely anonymized. A three-level quality control system, including self-inspection done by each center, inspection on all centers done by the leading unit, and spot-check auditing done by an independent third party (at the beginning and interim of the study), will be used to further guarantee the quality of the study. To improve patient compliance, research assistants regularly remind participants to receive treatment or assessment via telephone or WeChat. For participants who discontinue or deviate from intervention protocols, we will record the causes and clinical outcomes as much as possible.

## Discussion

The objective of this study is to validate the effectiveness of moxibustion for KOA, and to determine whether the habitat of moxa floss contributes to the effect of moxibustion.

In this trial, we chose KOA as the study disease because it is among those for which the effectiveness of moxibustion has been extensively tested. Also, the alleviation of pain – a key outcome measure for KOA – takes a shorter trial period than other outcome measures to reflect treatment effects. To better observe the between-group differences, we will only enroll participants with moderate to severe KOA.

A three-armed, randomized, controlled trial design including two types of comparison is applied in this trial. The comparison between real and sham moxibustion is to validate the effectiveness of moxibustion for KOA patients; the comparison between two habitats of moxa-stick moxibustion is to demonstrate the correlation between the moxa habitat and the moxibustion effect. For the moxa habitat comparison, we have chosen the TCM-acknowledged optimal habitat (Qichun County, Hubei Province, China) and another large habitat for commercially available moxa products, but which is not recognized by TCM theory (Nanyang County, Henan Province, China). By comparing these two habitats, we aim to make clear whether the habitat of moxa contributes to the effect of moxibustion or not.

The commonly used method for sham or placebo moxibustion is heat-insulated moxibustion using the same moxa material as the real moxibustion [25, 26]. This kind of sham control helps to show the contribution of heat, not the material, to moxibustion. In this trial, we will focus on the part that the material plays in moxibustion. The non-moxa stick, which is made from the leaves of the Artemisia capillaris Thunb, a Chinese herb that belongs to the same family and genus with Artemisia argyi (Artemisia L., Compositae), and shares the same appearance and process with the moxa stick, will be used for sham control. A good patient-blinding effect was achieved in the pre-study assessment. Though it is hard to distinguish two habitat moxa stick moxibustion, the blinding of the practitioner was negatively affected due to a less fragrant odor, looser ashes, and more smoke of the sham moxibustion. However, the failure of practitioner blinding cannot significantly affect the results of this trial, since the independent staff will be responsible for the assessment of the treatment effect. Also, this is a new attempt to provide a kind of blinding method for moxa stick moxibustion.

There are limitations in this study. The sample size lacks strong support for the difference between moxibustion using different habitat moxa floss. The trial duration is short (a 2-week treatment and a 4-week follow-up), since we focus on the short-term analgesic effect of moxibustion. We will not measure the change in the knee joint effusion volume, an objective outcome for KOA, due to the short treatment period. The blindness of the practitioner partly failed in the pre-study assessment, which may bring bias.

In conclusion, results of this trial will show the effect differences of moxibustion between real and sham moxibustion and between moxibustion using different habitat moxa floss, and thus to answer whether moxa floss is a better moxibustion material, and whether the habitat of moxa contributes to the effect of moxibustion. This study will contribute to the research of moxibustion worldwide via publishing results in a peer-reviewed journal (Additional file 1).

### Trial status

This trial is currently recruiting participants.

## Additional file

Additional file 1: SPIRIT 2013 Checklist. (DOC 128 kb)

## Abbreviations

AEs: Adverse events
ANCOVA: Analysis of covariance
KOA: Knee osteoarthritis
NSAIDs: Nonsteroidal anti-inflammatory drugs
OA: Osteoarthritis
OMERACT-OARSI: Outcome Measures in Rheumatology Arthritis Clinical Trials-Osteoarthritis Research Society International
PGA: Patient global assessment
TCM: Traditional Chinese medicine
VAS: Visual analogue scale
WOMAC: Western Ontario and McMaster Osteoarthritis Index
